# 

**Published:** 1827-01

**Authors:** 


### LONDON MEDICAL AND PHYSICAL JOURNAL.

EDITED BY

RODERICK MACLEOD, M.D.

PHYSICIAN TO THE
WESTMINSTER GENERAL DISPENSARY,
AND LECTURER
ON THE THEORY AND PRACTICE OF PHYSIC, AND ON MATERIA MEDICA,
AT THE SCHOOL IN GREAT WINDMILL STREET.

(VOL. LVII.)

NEW SERIES, VOL. II.

*Et quoniam variant morbi, variabimus artes;*

*Mille mali species, mille salutis erunt.*

LONDON:

JOHN SOUTER, 73, ST. PAUL'S CHURCH-YARD.

AND TO BE HAD OF ALL THE MEDICAL BOOKSELLERS.

1827.

Surgeon Genl's Office LIBRARY 45570

W1
LD 31

*J. and C. Adlard, Printers,*
*Bartholomew Close.*

### MONTHLY LIST OF MEDICAL BOOKS.

[*No books can be entered on this List except those sent to us for the purpose; as, in the list hitherto transmitted, the names of works have frequently been given as published, which have not appeared for weeks, or even months, after.*]

Materia Indica, or some Account of those Articles which are employed by the Hindoos, and other Eastern Nations, in their Medicine, Arts, and Agriculture: comprising also a Formulæ, with Practical Observations, Names of Diseases in various Eastern Languages, and a copious List of Oriental Books immediately connected with general Science, &c. &c. By Whitelaw Ainslie, m.d. m.r.a.s. late of the Medical Staff of Southern India.—2 vols. 8vo. London, 1826.

A New Supplement to the Pharmacopœias of London, Edinburgh, Dublin, and Paris; forming a complete Dispensatory and Conspectus; including the new French Medicines, as well as Herbs, Drugs, Compounds, Veterinary Drugs, Patent Medicines, Perfumery, Paints, Varnishes, and similar Articles kept in the Shops; with their Composition, Uses, Doses, and Adulterations; being a general Book of Formulæ for daily Reference in the Laboratory, and at the Counter. By James Rennie, a.m. Surgeon.—8vo. London, 1826.

A Treatise on Desk Diseases; containing the best Methods of Treating the various Disorders attendant upon Sedentary and Studious Habits, with a variety of Prescriptions adapted to each particular Affection. By W. M. Wallace, m.r.c.s.—8vo. London, 1826.

An Introductory Lecture on Human and Comparative Physiology, delivered at the New Medical School in Aldersgate-street. By Peter M. Roget, m.d. f.r.s. &c.—8vo. London, 1826.

An Introductory Lecture on Anatomy, delivered at the New Medical School in Aldersgate-street, Oct. 2, 1826. By Frederick Tyrrell, Surgeon to St. Thomas's Hospital and to the London Ophthalmic Infirmary.—8vo. London, 1826.

Practical Observations on the Teeth and Gums, with the best Mode for their Preservation. Dedicated by permission to John Alderson, Esq. m.d. by I. L. Levison, Surgeon-dentist, late of 54, Berwick-street, Oxford-street.—8vo. London, 1826.

### TO THE READERS OF THE MEDICAL AND PHYSICAL JOURNAL.

*The first volume of the New Series has now been completed, and the Editor cannot suffer the opportunity to pass without expressing his sense of obligation to those Gentlemen by whose co-operation and support he has been enabled to carry the proposed plan into effect.*

*The principle of preserving an authentic record of the most interesting* Cases *occurring at* Public Institutions, *and giving a correct account of the* Observations *made by the Medical Officers, was obviously good; but it remained to be ascertained how far those Gentlemen, who had in the first instance consented to the arrangement, would persevere under the misrepresentation and obloquy which it was anticipated, as a matter of course, would be heaped upon them. This circumstance alone rendered the undertaking one of some anxiety to the Editor. The experiment, however, has proved eminently successful: the venom of the shaft is no longer mistaken for the strength of the bow; and the Editor has had the gratification to find that the Contributions to the Original Department, so far from having diminished, have greatly increased both in number and value since the commencement of the New Series.*

*It may be proper to state with regard to the Cases from Hospitals and other Public Institutions, that either the MS. or the proof-sheets have been revised by the Gentlemen whose observations have been detailed; the Editor is therefore persuaded that no material inaccuracy will be found in these Reports, in which is recorded the experience of some of the most eminent Practitioners in the metropolis.*

*It may not be irrelevant to add, that the Editor is informed by the Proprietors that the change has likewise been highly beneficial to them; the sale of the Journal having progressively increased since July; a circumstance gratifying to the Editor, as it may fairly be regarded as showing that the arrangements adopted in the New Series have met with the approbation of his professional brethren.*

### NOTICES.

*After a careful perusal of* Dr. N.'s *Paper on the* "Source and Identity of the Principle of Animal Life," *we feel ourselves obliged to decline it, as of a nature too speculative for this Journal.*

*Communications have reached us from* Mr. Cleghorn, Mr. Abrahams, *and* Dr. Bow.

*Numerous Papers have been received from Practitioners in London; as these are privately acknowledged, we do not think it necessary to announce them publicly.*

